# The Loss of Efficiency Caused by Agents’ Uncoordinated Routing in Transport Networks

**DOI:** 10.1371/journal.pone.0111088

**Published:** 2014-10-28

**Authors:** Zhongzhi Xu, Li Sun, Junjie Wang, Pu Wang

**Affiliations:** School of Traffic and Transportation Engineering, Central South University, Changsha, Hunan, P.R. China; Beihang University, China

## Abstract

Large-scale daily commuting data were combined with detailed geographical information system (GIS) data to analyze the loss of transport efficiency caused by drivers’ uncoordinated routing in urban road networks. We used Price of Anarchy (POA) to quantify the loss of transport efficiency and found that both volume and distribution of human mobility demand determine the POA. In order to reduce POA, a small number of highways require considerable decreases in traffic, and their neighboring arterial roads need to attract more traffic. The magnitude of the adjustment in traffic flow can be estimated using the fundamental measure traffic flow only, which is widely available and easy to collect. Surprisingly, the most congested roads or the roads with largest traffic flow were not those requiring the most reduction of traffic. This study can offer guidance for the optimal control of urban traffic and facilitate improvements in the efficiency of transport networks.

## Introduction

In this era of unprecedented global urbanization, the fast growth of human mobility has put immense pressure on urban roads [1]–[3], which has manifested in the form of severe traffic congestion and traffic-related air pollution [4]–[7]. Improving the efficiency of transport networks has become an urgent problem to solve and it has recently attracted widespread attention from scientific and engineering fields [8]–[14]. The transport efficiency of a road network is primarily determined by its network topology [15]–[21], by the volume and distribution of travel demand [9], [18]–[20], and by drivers’ routing behavior (the manner in which the road network is used) [11]–[14]. Studies have shown that increasing the capacity of the important backbone of transportation networks [10] or removing specific segments [11] can make these networks more efficient. It has also been discovered that traffic congestion can be mitigated efficiently by intelligently reducing a small fraction of travel demand [9].

Despite the intensive investigations on the effects of network topology and travel demand on the efficiency of transport networks, the effect of agents’ routing behavior has only been studied on theoretical networks or simplified road networks, without considering actual travel demand [11]–[15]. Using three urban road networks, one type of transport networks, as an example, we show a comprehensive image of the loss of transport efficiency caused by agents’ (drivers’) uncoordinated routing. Furthermore, we explored the way that can lead a road network to its optimal state. We also believe our findings can shed light on improving other types of transport networks experiencing a lack of coordination among agents, such as the Internet [22].

## Data and Methods

**Figure 1:**
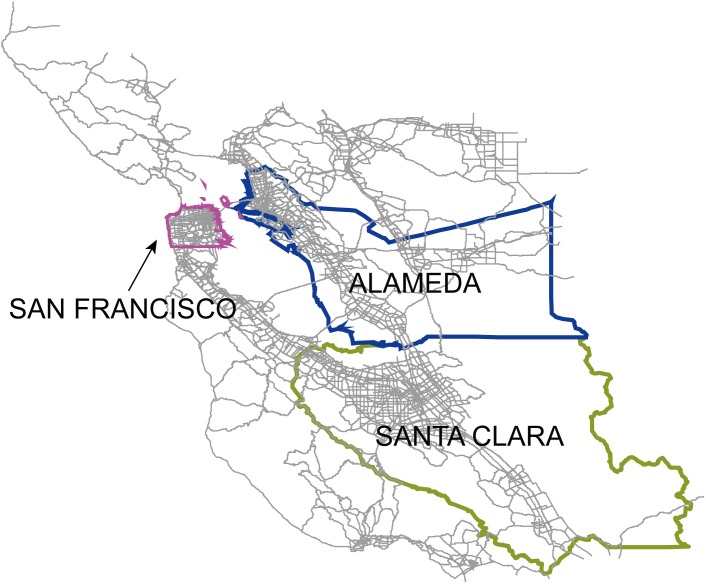
Bay Area road network and locations of three counties.

The road networks in San Francisco, Santa Clara, and Alameda were extracted from the Bay Area road network (Figure 1), which was provided by NAVTEQ, a commercial provider of geographical information systems data [23]. The road networks are composed of highways and arterial roads. There are 2,816 road segments in San Francisco, 7,269 in Santa Clara, and 5,805 in Alameda. There are 1,144 intersections in San Francisco, 3,420 in Santa Clara, and 2,744 in Alameda. More detailed information regarding the three road networks was provided in Table S1 and Figure S1.

In the following, the Bay Area commuting OD (origin-destination matrix) was first estimated, and consequently the commuting OD was calculated for each county. The Bay Area daily home-work commuting data were provided by the U.S. census bureau [24]. The numbers of trips from residents’ home locations to work locations at a street-block level were recorded. The street blocks were grouped into census tracts where they were located (1,398 in total) for generating the census tract based OD.

People use various modes of transportation in their daily commutes, these include cars (driving alone), carpooling, public transportation, bicycling, and walking. Based on the mode split data, the vehicle use rate was calculated for each census tract [25]:
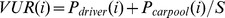
(1)Here, 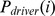 and 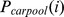 are the fractions of residents in census tract *i* who drive alone or share a car. The average carpool size in California 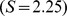 was used for these calculations [26]. Next, a mode of transportation (vehicle or non-vehicle) was randomly assigned to each of the residents of each census tract according to the estimated vehicle usage rate 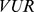 (Figure S2). Then the trips not completed by vehicles were filtered out, generating the vehicle-based commuting OD.

The average number of daily trips per person is about 4 in the U.S., this generates about 22 million trips in the Bay Area [27]. Based on the daily distribution of traffic volume, average hourly trip production 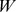 during the morning commute (6∶00 a.m.–10∶00 a.m.) was estimated [28]. The vehicle-based daily commuting OD was rescaled using 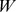 to estimate the morning peak hourly commuting OD.

To assign trips to the road networks, the census tract based OD was mapped to the intersection-based OD. For each trip in the census tract based OD, the road intersections within the origin census tract and destination census tract were identified (Figure S3). One intersection in the origin census tract and one intersection in the destination census tract were randomly selected as the origin and destination of the trip in the intersection-based OD.

**Figure 2:**
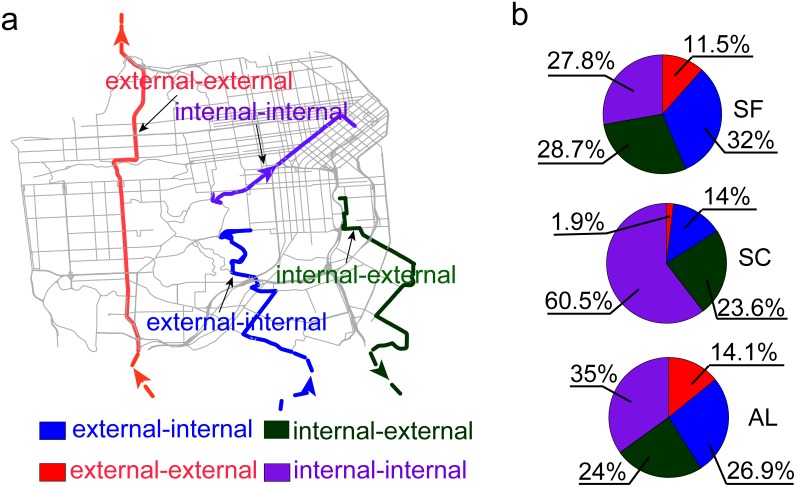
Illustration and statistics of the four types of trips. (*a*) Four types of trips defined by the locations of the origin and destination. Purple, green, blue, and red lines represent the path of an internal-internal trip, an internal-external trip, an external-internal trip, and an external-external trip respectively. (*b*) The statistics of the four types of trips in San Francisco (SF), Santa Clara (SC) and Alameda (AL) (also see Table S2).

Finally, the commuting ODs for the three road networks were extracted. Using San Francisco as an example, trips start and end within the county formalize the internal-internal OD, trips that start within the county and end outside it and trips start outside the county and end within it formalize the internal-external OD and the external-internal OD. Trips that start and end outside the county formalize the external-external OD. The origins and destinations of each internal-external trip, external-internal trip, and external-external trip were mapped to the San Francisco road network (Figure 2a). For each internal-external trip, the Dijsktra algorithm was used to find the shortest path (measured in travel time) within the Bay Area road network [29]. The destination of the trip was replaced with the last road intersection that the driver passed before leaving San Francisco. For each external-internal trip, the origin of the trip was mapped to the first road intersection that the driver passed upon entering San Francisco. For each external-external trip, the new origin and destination were mapped to the first road intersection that the driver passed upon entering San Francisco and the last intersection passed before leaving San Francisco. The four types of ODs were also generated for Santa Clara and Alameda.

Due to different geographic locations and patterns of land use, the four types of ODs showed different combinations in the three counties (Figure 2b). In San Francisco, there were similar numbers of internal-internal trips, internal-external trips, and external-internal trips. There were slightly more external-internal trips than internal-external trips, indicating that more people enter San Francisco during the morning peak. In Santa Clara, the majority of trips (60.5%) were within the county. There were many more people leaving than entering the county during the morning. People rarely drove across the county. In Alameda, although the majority of trips began and ended within the county, the number of internal-internal trips (35%) was only slightly higher than that of internal-external trips (24%) and external-internal trips (26.9%). Alameda had the most cross-county trips.

**Figure 3:**
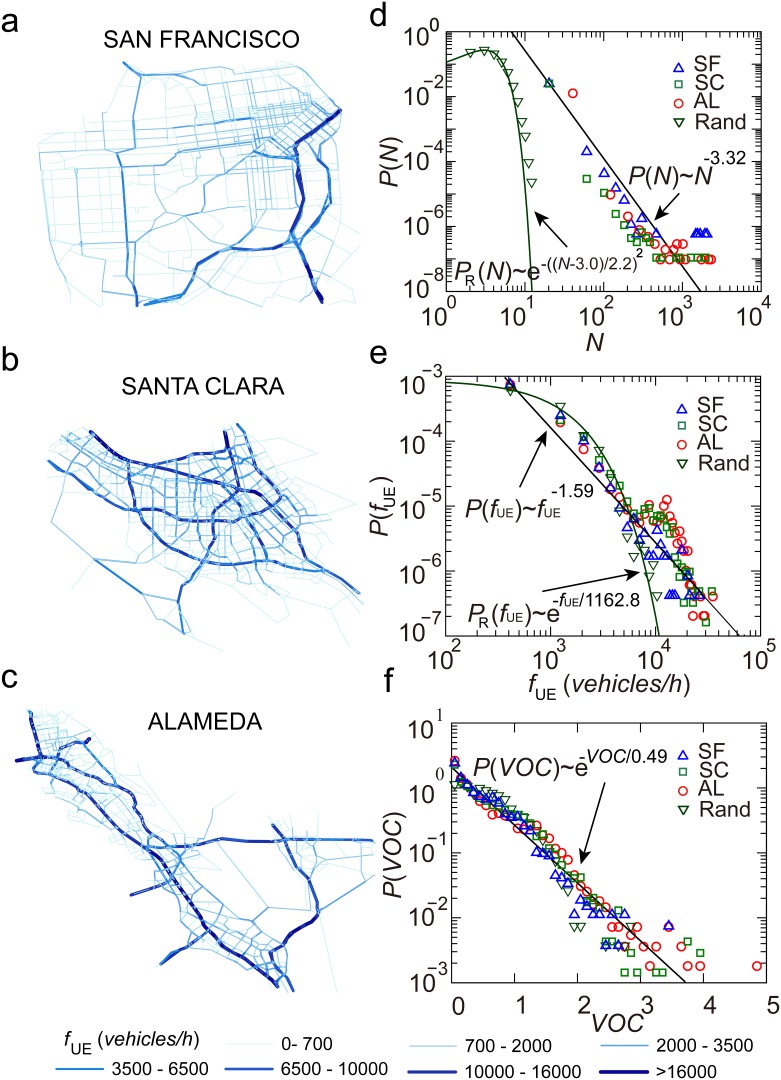
Statistical analysis of traffic flow and volume over capacity (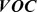). (*a*), (*b*), (*c*) Spatial distributions of traffic flow are shown for San Francisco (SF), Santa Clara (SC), and Alameda (AL). (*d*) The number of trips between a pair of origin and destination. (*e*) Traffic flow 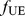 was estimated using the actual ODs and the random OD. (*f*) 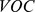 was estimated using the four ODs.

The number of trips between a pair of origin and destination 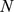 can be approximated by a power-law distribution 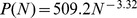 for all the three counties 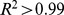, showing that travel demand between most pairs of locations was small, but there was high volume between a few origins and destinations (Figure 3d). The random OD, which had the same number of OD pairs and the same number of trips as the San Francisco OD, was generated and used for conducting comparative studies. The trips in the random OD were randomly assigned to pairs of origins and destinations.

## User Equilibrium and Social Optimum

Based on the ODs generated for the three road networks, traffic flow along each road segment was estimated under two scenarios. In the first scenario, all drivers were assumed to know all information regarding the road network and put their own interests first. In this scenario, the whole system reached the user equilibrium (UE) such that no driver could reduce travel time any further by switching paths. This is also known as the Nash equilibrium [12]. The second scenario was the social optimum (SO), the state that was most beneficial overall, meaning that it minimized the total travel time across the whole system [11], [30]. The price of anarchy POA was then defined as the ratio of the total travel time of the Nash equilibrium and the total travel time of the social optimum:
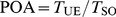
(2)


The price of anarchy POA quantifies the loss of transportation efficiency caused by drivers’ selfish routing. Given the huge traffic volume in a big city, a small POA can still mean a big loss of efficiency. Understanding the method to reduce POA has important consequences for the optimal design and control of transportation systems.

To calculate the equilibrium flow 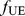 under UE scenario, Wardrop’s principle, as described by the Beckmann model, was used [31]. In this case, the objective function in Eq. (3) was minimized:
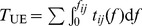
(3)Here, 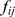 and 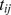 represent the traffic flow and travel time between a pair of road intersections *i* and *j*. Travel time 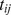 was estimated using the Bureau of Public Roads (BPR) function, which is widely used in civil engineering:
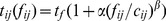
(4)Here, 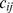 is the capacity of the road segment from road intersection *i* to road intersection *j*. Commonly used values 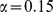 and 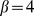 were selected [25]. The equilibrium flows 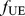 were then numerically calculated using the Frank-Wolfe algorithm (see Method S1) toolkit provided by TransCAD 5.0, a transportation planning software [25], [32].

Under the SO scenario, the socially optimal traffic flows 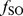 were estimated by minimizing total travel time in Eq. (5):
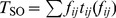
(5)


To calculate the socially optimal flow 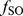, the function of travel time was converted to the following:
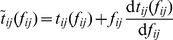
(6)


Computing the integral of 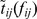, the following was determined:
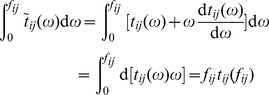
(7)

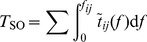
(8)


In this way, equation (7) possesses the same form as equation (3). Traffic flow 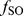 can also be numerically calculated using the Frank-Wolfe algorithm toolkit with the travel time function 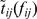 (Eq. (6)).

## Results

Experimental tests show that humans find paths within networks in the perspective of minimizing their own travel costs [33], [34]. For this reason, the equilibrium flow 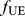 was used to analyze road usage patterns (Figure 3a–c). As Figure 3e shows that the equilibrium flow 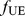 can be well approximated by a power-law distribution 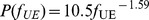 for San Francisco, Santa Clara, and Alameda, indicating that road usages are similar and heterogeneous in the three counties (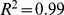). Traffic flow for more than 80% of the road segments was below 2,000 (*vehicles/hour*), and there were 1.0%, 3.4%, and 5.6% of the road segments in San Francisco, Santa Clara, and Alameda having their 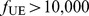 (*vehicles/hour*). The equilibrium flow 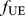 was also measured using the random OD of San Francisco and observed to follow an exponential distribution 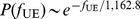 (Figure 3e). This indicates a much faster decay.

The volume over capacity 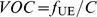 was also measured (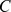 is the capacity of a road segment). As shown in Figure 3f, 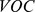 estimated using either the actual ODs or the random OD can be approximated using an exponential distribution 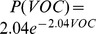 (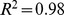), showing traffic flow in most road segments to be well within the roads’ capacities, but there was a small number of congested road segments.

**Figure 4:**
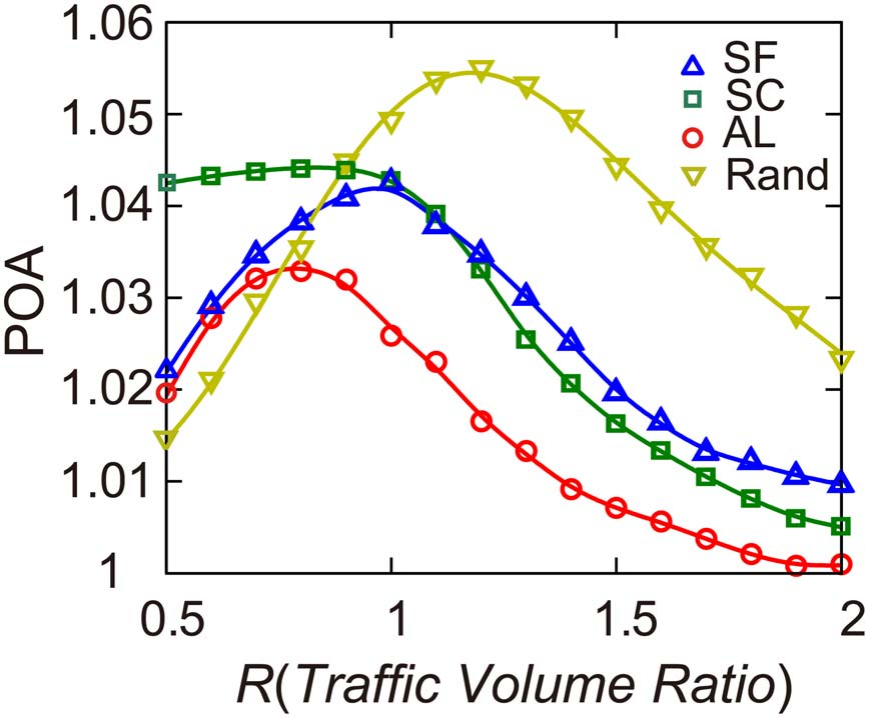
Price of anarchy (POA) versus traffic volume ratio *R*. Using actual travel demand distributions, the maximum POA = 1.043 and POA = 1.033 were observed at *R* = 0.8 for Santa Clara and Alameda. In San Francisco, the maximum POA estimated using actual travel demand distributions and random travel demand distributions were observed at *R* = 1.0 (POA = 1.041) and *R* = 1.2 (POA = 1.056) respectively.

To determine how varying traffic volumes (total number of trips) affect POA, traffic volume ratio *R* was defined as the potential traffic volume over the current traffic volume. Traffic volumes of the actual ODs and the random OD were scaled up or down using parameter *R*, at the same time the original distributions of travel demands were kept. As shown in Figure 4, the maximum POA = 1.043 and POA = 1.033 were both observed at *R* = 0.8 in Santa Clara and Alameda, and the maximum POA estimated using actual distribution of travel demand was 1.041 in San Francisco when *R* = 1.0 (Table S3). All of the POA first increased with *R* and then decreased with *R*, suggesting that the traffic flow patterns are similar under UE and SO scenarios when traffic volume is very small or very large. This validates the generality of the findings obtained in [11]. Because when employing more-detailed travel demand information and road network information, similar pattern of POA versus traffic volume was still observed.

In Figure 4, the random OD of San Francisco was used to show how distribution of travel demand determines the POA. Unlike the maximum POA, which was predicted using the actual OD, a larger maximum POA = 1.056 was observed at a larger traffic volume ratio *R* = 1.2. POA was estimated using the actual OD and the random OD, which were 1.021 and 1.015, respectively, for small values of *R*, such as *R* = 0.5. They were 1.01 and 1.023, respectively, for large values of *R*, such as *R* = 2.0. The different patterns observed for POA versus *R* in these three counties also confirm that the distribution of travel demand needs to be considered in estimating the price of anarchy.

**Figure 5:**
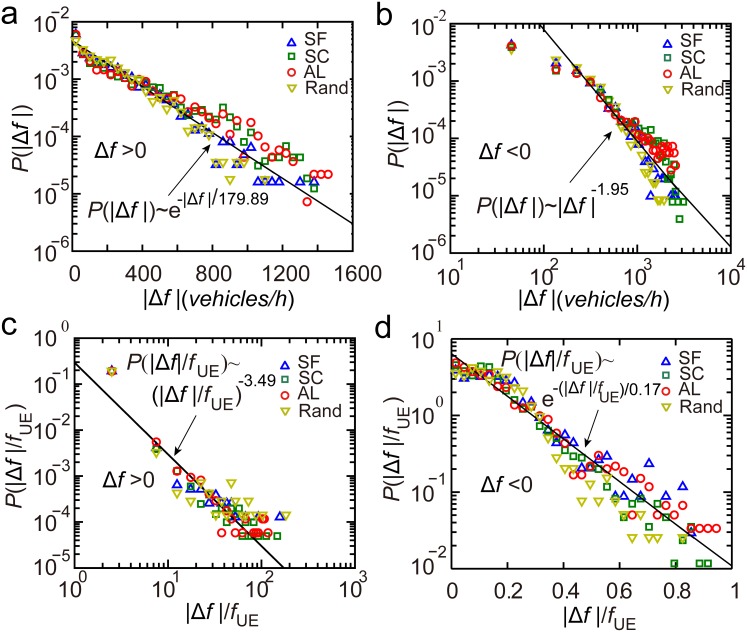
Difference between equilibrium flow 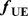 and socially optimal flow 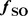. (*a*), (*b*) The difference between equilibrium flow and socially optimal flow 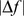 in the cases of 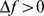 and 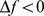. (*c*), (*d*) The rescaled difference between equilibrium flow and socially optimal flow 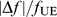 in the cases of 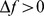 and 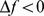.

Given the huge traffic volumes in urban areas, a small POA can still mean a big loss of transport efficiency. Taking San Francisco as an example (POA = 1.041), drivers’ selfish routing produced 1073.3 hours more travel time during only one hour of the morning rush. To offer guidance to reduce POA in urban road networks, the differences in equilibrium flow and socially optimal flow 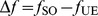 were measured for each road segment. Results showed 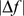 to be heterogeneously distributed in the road networks (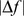 = 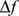 when 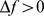 and 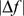 = 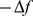 when 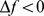). Most road segments have a small 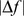 and a few road segments, the targets of urban traffic controls, have 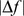 values as large as 3,000 (*vehicles/hour*). In the three counties, distributions of 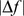 can be approximated by an exponential distribution 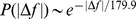 (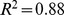) when 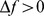 and by a power-law distribution 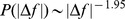 (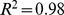) when 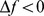 (Figure 5a and b). The rescaled traffic flow difference 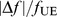, which quantifies the relative difference between 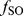 and 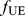, can be approximated by a power-law distribution 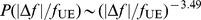 (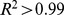) when 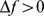 and by an exponential distribution 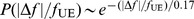 (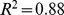) when 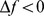 (Figure 5c and d).

**Figure 6:**
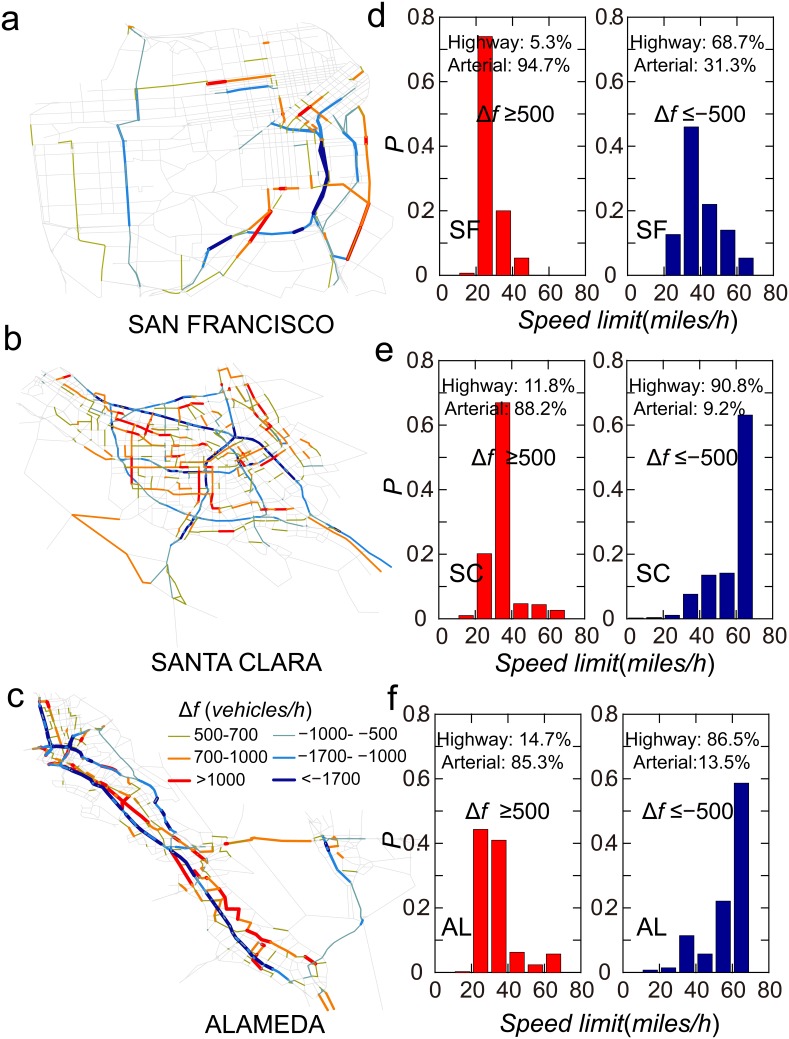
Illustration of road segments with large 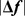. (*a*), (*b*), (*c*) Road segments with 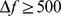 (*vehicles/hour*) and road segments with 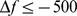 (*vehicles/hour*) in San Francisco, Santa Clara, and Alameda. (*d*), (*e*), (*f*) Speed limits of road segments with 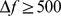 (*vehicles/hour*) and 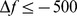 (*vehicles/hour*) in San Francisco, Santa Clara, and Alameda.

**Figure 7:**
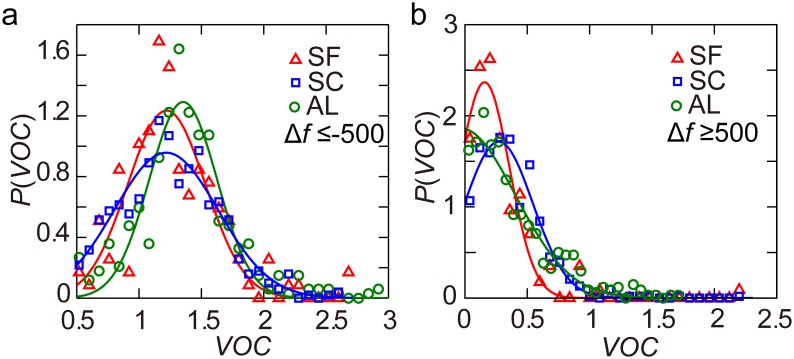
Volume over capacity (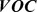) of road segments with large 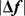. (*a*) 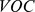 of road segments with 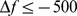 (*vehicles/hour*) can be approximated by Gaussian distributions 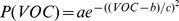 with *a* = 1.24 (0.96, 1.29), *b* = 1.22 (1.22, 1.35), *c* = 0.44 (0.59, 0.38), and 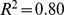 (0.92, 0.89) for San Francisco (Santa Clara, Alameda). (*b*) 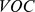 of road segments with 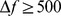 (*vehicles/hour*) can be approximated by Gaussian distributions 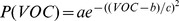 with *a* = 2.37 (1.72, 1.86), *b* = 0.16 (0.27, −0.04), *c* = 0.28 (0.39, 0.62), and 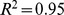 (0.97, 0.95) for San Francisco (Santa Clara, Alameda).

The heterogeneously distributed 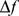 suggests that traffic flow in only a small number of roads requires considerable adjustment. We found only 5.3% of the road segments in San Francisco, 9.3% of those in Santa Clara, and 9.8% of those in Alameda had 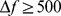 (*vehicles/hour*) or 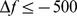 (*vehicles/hour*) (Figure 6a–c, Table S3). In San Francisco, Santa Clara, and Alameda, 94.7%, 88.2%, and 85.3% of the road segments with 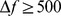 (*vehicles/hour*) are arterial roads, and 68.7%, 90.8%, and 86.5% of the road segments with 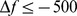 (*vehicles/hour*) are highways (Figure 6d–f). Targeted highways can noticeably reduce travel time, so they attracted large amounts of traffic and were overly used (most of them had 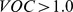) (Figure 7a). Targeted arterial roads near the targeted highways may be suitable as alternative paths, but they do not see as much use as they could (most of them had 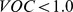) (Figure 7b). The present findings held true for all the three counties, indicating a general explanation for the price of anarchy in road networks. These results can provide insight that can be used to identify roads in need of traffic control in practical situations.

**Figure 8:**
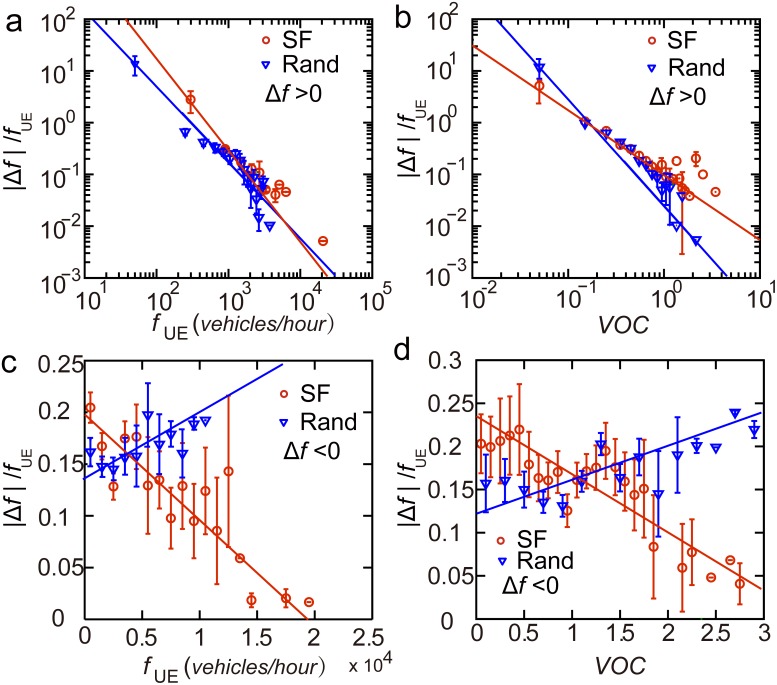
The magnitude of traffic flow adjustment for reducing POA. (*a*), (*b*) When increasing traffic flow of a road segment to reduce POA (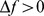), 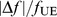 can be approximated by two power-law functions 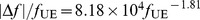 (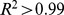) or 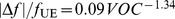 (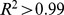). (*c*), (*d*) When decreasing traffic flow of a road segment to reduce POA (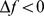), 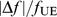 can be approximated by 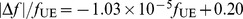 (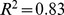) and 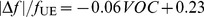 (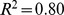). Error bar here represents a 95% confidence interval.

We next explored the magnitude of traffic flow adjustment to reduce POA. Taking San Francisco as an example, when increasing traffic flow of a road segment (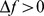), the rescaled difference of traffic flows 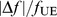 can be approximated by power-law functions 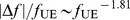 and 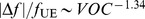. This offers a guidance for increasing traffic flow in the targeted road segments when their 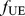 or 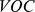 is known (Figure 8a and b). The increase of traffic flow in congested roads or roads with large traffic would be very tiny. Obvious increases in traffic flow were only observed in roads with low 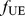 and 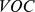. This law also holds for the case of the random OD.

When decreasing traffic flow of a road segment to reduce POA (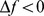), the rescaled difference of traffic flows 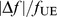 can be approximated by linear functions 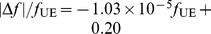 (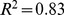) and 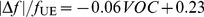 (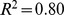) (Figure 8c and d). However, for the case of the random OD, different functional relationship was found, 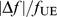 was observed to increase linearly with 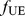 and 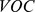 (Figure 8c and d).

**Figure 9:**
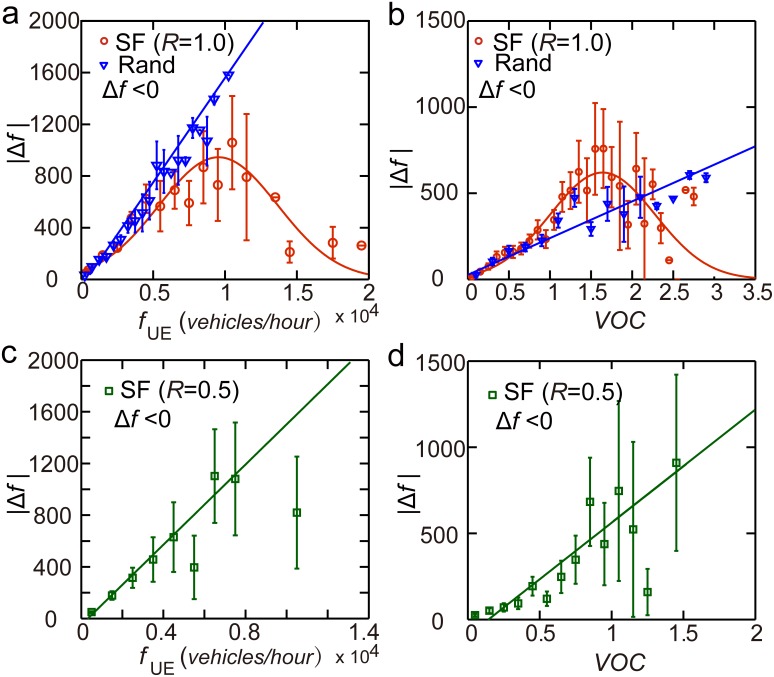
The distribution and volume of travel demand determines the adjustment of traffic flow. (*a*), (*b*) 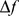 estimated using the San Francisco random OD and actual OD show different correlations with 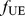 and 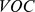. Statistical fits 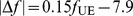 (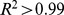), 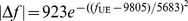 (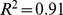), 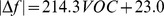 (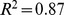) and 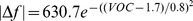 (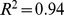) were plotted to guide the eyes. (*c*), (*d*) 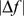 estimated using the re-scaled (*R* = 0.5) San Francisco actual OD. Statistical fits 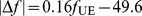 (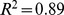) and 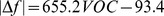 (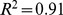) were plotted to guide the eyes. Error bar here represents a 95% confidence interval.

To understand the observed different relationships between 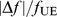 and 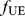 (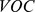), we analyzed the traffic flow difference 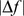 for the case of the random OD and the case of the actual OD. We found that 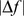 estimated using the random OD increased linearly with 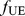 and 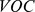. However, counter intuitively, 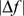 estimated using the actual OD first increased and then decreased with 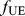 and 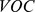. The roads with the largest flow or 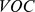 were not those requiring the highest reduction of traffic. The highest reduction of traffic flow was observed at intermediate values of 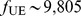 (*vehicles/h*) and 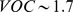 (Figure 9a and b).

The counter intuitive result could be resulted from the different distributions of traffic flow estimated by the random OD and the actual OD. Comparing with the case of the random OD, traffic flow estimated using the actual OD was more heterogeneously distributed. There are much more road segments with large traffic flow (Figure 3e), preventing drivers from selfishly switching routes. This phenomenon is similar to that when traffic volume is very large, traffic flow patterns under UE scenario and SO scenario are similar (POA∼1.0). To further test this explanation, the actual ODs were scaled down using parameter *R* = 0.5, the observed maximum traffic flow is largely reduced. As Figure 9c and d shows, the difference in traffic flow 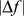 shows similar pattern with the case of the random OD rather than the case of the actual OD.

**Figure 10:**
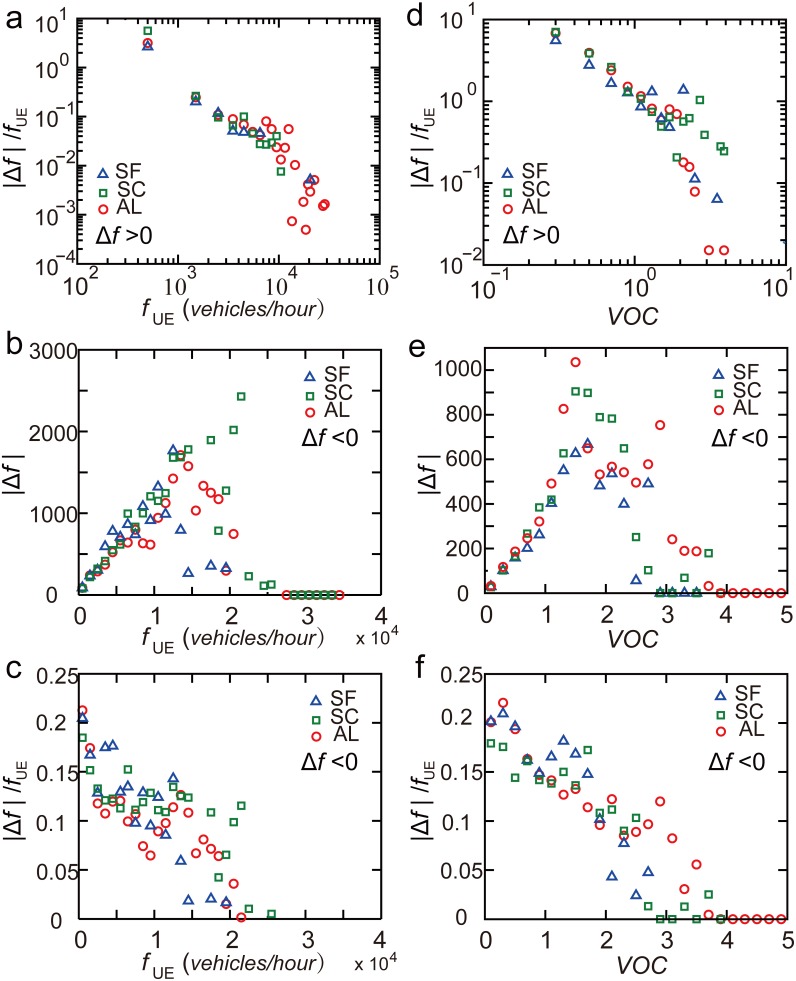
The magnitude of traffic flow adjustment for reducing POA in the three counties. (*a*) 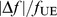 versus 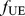 when 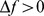. (*b*) 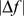 versus 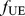 when 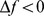. (*c*) 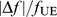 versus 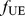 when 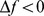. (*d*) 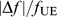 versus 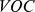 when 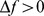. (*e*) 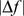 versus 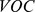 when 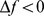. (*f*) 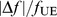 versus 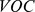 when 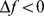.

We further analyzed the magnitude of traffic flow adjustment for reducing POA for Santa Clara and Alameda. Similar functional relationships between 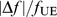 and 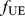, 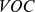 were observed (Figure 10). The significance of these findings is that it offered useful guidance that could reduce POA for urban road networks, using traffic flow 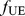 only. This was widely available and recorded by many devices.

## Conclusions

To conclude, we generate morning-peak commute ODs for three Bay Area counties and study the price of anarchy using actual travel demand in large-scale road networks. The different patterns observed for POA versus *R* in the three counties showed that both volume and distribution of travel demand determine the POA in a road network. The difference of equilibrium flow and socially optimal flow 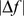 was measured for each road segment. It was found that 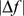 for most roads is tiny. For this reason, adjusting traffic flow of a small number of roads can push a system toward its social optimum. Here, roads with large 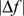 were found to be only a few highways that saw excessive use due to their high speed limits and the neighboring arterial roads, which can offer alternative paths. The rescaled traffic flow difference 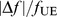 was found to have power-law functional relationships with 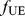 and 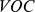 when 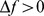 and have linear functional relationships with 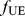 and 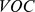 when 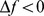. Surprisingly, the roads with the largest traffic flow and 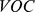 did not have the largest adjustment of traffic flow.

The present work can inform specific intervention strategies on reducing the loss of efficiency caused by agents’ selfish routing in urban road networks, a crucial infrastructure that billions of people use every day. The present work uses real-world travel demand data to explore the way to reduce POA. The elucidation of these findings may draw more attention to the use of actual transport demand information in the optimal control of a broad set of networks experiencing a lack of coordination among agents.

## Supporting Information

Figure S1 **Distributions of lengths and free travel times of road segments.** Both lengths and free travel times of road segments follow similar distributions in three counties.
(PDF)

Figure S2 **Vehicle usage rates (** ***VUR*** **) in census tracts.** Different colors represent different vehicle usage rates. Urban areas have lower *VUR* than suburban areas.
(PDF)

Figure S3 **Illustration of census tracts and convention from census tract based OD to intersection based OD.** The road segments in the vicinity of San Francisco downtown are depicted by gray lines and the small black dots are the road intersections that lie in the census tracts inside.
(PDF)

Table S1 **Census data and road networks in San Francisco, Santa Clara, and Alameda.**
(PDF)

Table S2 **Statistics of four types of trips in San Francisco, Santa Clara, and Alameda.**
(PDF)

Table S3 **A summarization of the results in** **figure 4** **and** **figure 6** **for San Francisco, Santa Clara, and Alameda.**
(PDF)

Method S1 **Solving the Beckmann model by the Frank-Wolfe algorithm.**
(PDF)
