# Supplementary material for: The Loss of Efficiency Caused by Agents’ Uncoordinated Routing in Transport Networks
Source: PLoS One. 2014 Oct 28;9(10):e111088. doi: 10.1371/journal.pone.0111088 (PMC4211890; doi:10.1371/journal.pone.0111088)
Supplement: Figure S1 — Distributions of lengths and free travel times of road segments. Both lengths and free travel times of road segments follow similar distributions in three counties. (PDF) [file pone.0111088.s001.pdf]

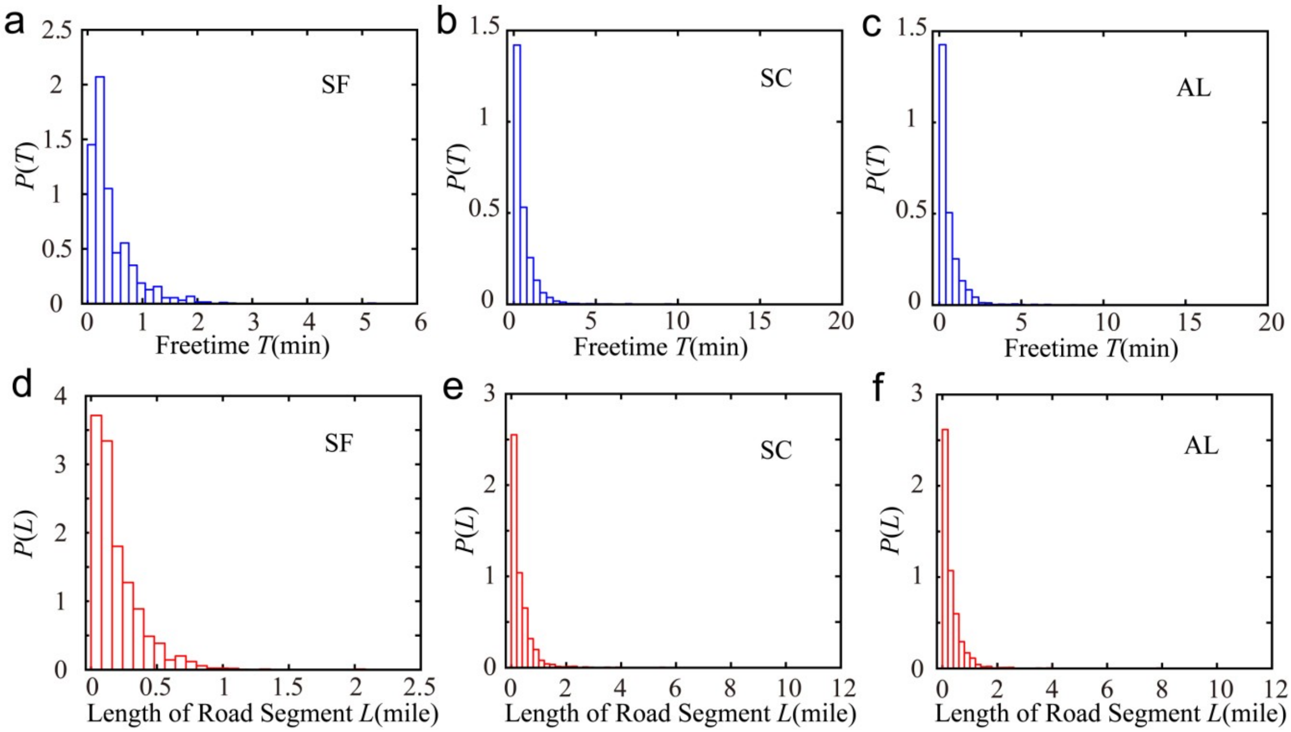

Figure S1. Distributions of lengths and free travel times of road segments. Both lengths and free travel times of road segments follow similar distributions in three counties.
