# Supplementary material for: The Loss of Efficiency Caused by Agents’ Uncoordinated Routing in Transport Networks
Source: PLoS One. 2014 Oct 28;9(10):e111088. doi: 10.1371/journal.pone.0111088 (PMC4211890; doi:10.1371/journal.pone.0111088)
Supplement: Figure S2 — Vehicle usage rates ( VUR ) in census tracts. Different colors represent different vehicle usage rates. Urban areas have lower VUR than suburban areas. (PDF) [file pone.0111088.s002.pdf]

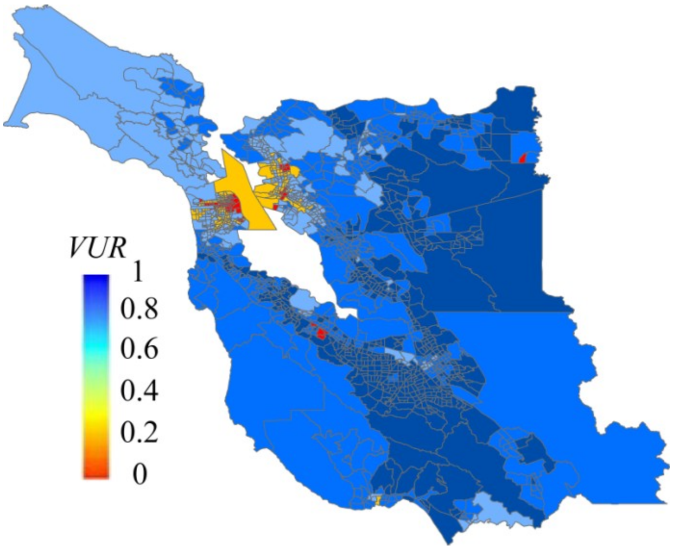

Figure S2.Vehicle usage rates (*VUR*) in census tracts. Different colors represent different vehicle usage rates. Urban areas have lower *VUR* than suburban areas.
