# Supplementary material for: The Loss of Efficiency Caused by Agents’ Uncoordinated Routing in Transport Networks
Source: PLoS One. 2014 Oct 28;9(10):e111088. doi: 10.1371/journal.pone.0111088 (PMC4211890; doi:10.1371/journal.pone.0111088)
Supplement: Figure S3 — Illustration of census tracts and convention from census tract based OD to intersection based OD. The road segments in the vicinity of San Francisco downtown are depicted by gray lines and the small black dots are the road intersections that lie in the census tracts inside. (PDF) [file pone.0111088.s003.pdf]

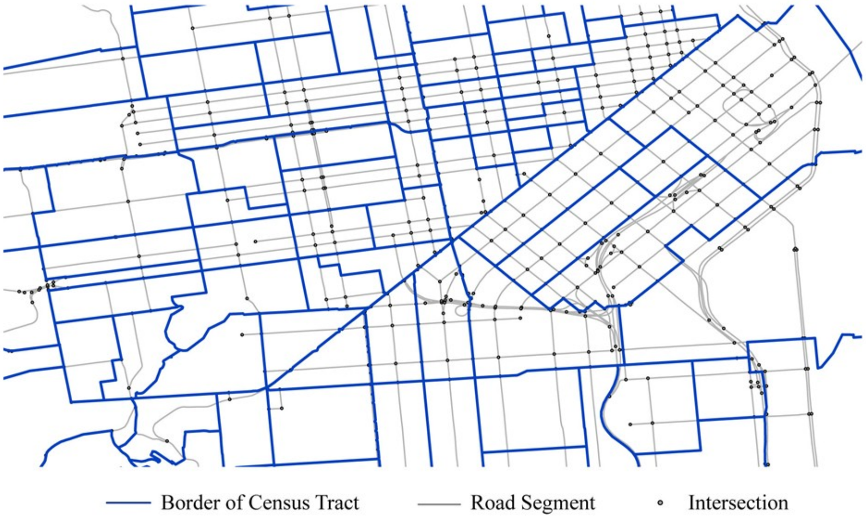

Figure S3. Illustration of census tracts and convention from census tract based OD to intersection based OD. The road segments in the vicinity of San Francisco downtown are depicted by gray lines and the small black dots are the road intersections that lie in the census tracts inside.
