# Supplementary material for: The Loss of Efficiency Caused by Agents’ Uncoordinated Routing in Transport Networks
Source: PLoS One. 2014 Oct 28;9(10):e111088. doi: 10.1371/journal.pone.0111088 (PMC4211890; doi:10.1371/journal.pone.0111088)
Supplement: Table S1 — Census data and road networks in San Francisco, Santa Clara, and Alameda. (PDF) [file pone.0111088.s004.pdf]

Properties	SF	SC	AL
**Population**	776,733	1,682,585	1,443,741
**Area (miles^2)**	47.54	1,298	743.97
**Population density (/miles^2)**	16,338.5	1,296.3	1,940.6
**Number of census tracts inside**	195	372	360
**Number of road segments**	2,816	7,269	5,805
**Number of road intersections**	1,144	3,420	2,744
**Avg. length of road segments (miles)**	0.20	0.33	0.33
**Avg. free travel time of road segments (minutes)**	0.43	0.58	0.60
**Number of vehicle trips in 1 hour of morning peak**	35,212	93,882	79,573
**Avg. estimated travel time per triph (minutes)**	11.18	22.54	31.61

Table S1. Census data and road networks in San Francisco, Santa Clara , and Alameda.
