# Supplementary material for: The Loss of Efficiency Caused by Agents’ Uncoordinated Routing in Transport Networks
Source: PLoS One. 2014 Oct 28;9(10):e111088. doi: 10.1371/journal.pone.0111088 (PMC4211890; doi:10.1371/journal.pone.0111088)
Supplement: Table S2 — Statistics of four types of trips in San Francisco, Santa Clara, and Alameda. (PDF) [file pone.0111088.s005.pdf]

Number	SF	SC	AL
**Internal-internal trips**	39,314	227,208	111,373
**Internal-external trips**	40,576	88,815	76,419
**External-internal trips**	45,215	52,711	85,648
**External-external trips**	16,391	7,081	44,936

Table S2. Statistics of four types of trips in San Francisco, Santa Clara, and Alameda.
