# Supplementary material for: The Loss of Efficiency Caused by Agents’ Uncoordinated Routing in Transport Networks
Source: PLoS One. 2014 Oct 28;9(10):e111088. doi: 10.1371/journal.pone.0111088 (PMC4211890; doi:10.1371/journal.pone.0111088)
Supplement: Table S3 — A summarization of the results in figure 4 and figure 6 for San Francisco, Santa Clara, and Alameda. (PDF) [file pone.0111088.s006.pdf]

Main Results	SF	SC	AL
**The maximum value of POA**	1.041	1.043	1.033
**The R value when POA reaches the maximum**	1.0	0.8	0.8
**Fraction of highways with $\Delta f \geq 500$ (vehicles/h)**	0.053	0.118	0.147
**Fraction of highways with $\Delta f \leq -500$ (vehicles/h)**	0.687	0.908	0.865
**Fraction of arterial roads with $\Delta f \geq 500$ (vehicles/h)**	0.947	0.882	0.853
**Fraction of arterial roads with $\Delta f \leq -500$ (vehicles/h)**	0.313	0.092	0.135

Table S3. A summarization of the results in figure 4 and figure 6 for San Francisco, Santa Clara, and Alameda.
