# Supplementary material for: The Loss of Efficiency Caused by Agents’ Uncoordinated Routing in Transport Networks
Source: PLoS One. 2014 Oct 28;9(10):e111088. doi: 10.1371/journal.pone.0111088 (PMC4211890; doi:10.1371/journal.pone.0111088)
Supplement: Method S1 — Solving the Beckmann model by the Frank-Wolfe algorithm. (PDF) [file pone.0111088.s007.pdf]

Solving the Beckmann model by the Frank-Wolfe algorithm

The Frank-Wolfe algorithm was proposed by Marguerite Frank and Philip Wolfe in 1956 [1]. It was first used by LeBlanc to solve the Beckmann model in 1975 [2], the detailed method is:

Step 1: Use the Dijsktra algorithm [3] to calculate the shortest path for each trip based on the free flow travel time t_f. Then calculate the traffic flow of each road segment $f_{ij}{}^n$, set *n*=1.

Step 2: Update the travel time of each road segment $t_{ij}(f_{ij}{}^n)$ by the BPR function.

Step 3: Use the Dijsktra algorithm to calculate the shortest path for each trip based on the updated travel times $t_{ij}(f_{ij}{}^n)$. Then calculate the traffic flow of each road segment $g_{ij}{}^n$.

Step 4: Use the bisection method to find the parameter λ satisfying:

$$\sum (g_{ij}{}^n - f_{ij}{}^n) \times t_{ij}\left(f_{ij}{}^n + \lambda\left(g_{ij}{}^n - f_{ij}{}^n\right)\right) = 0$$

Step 5: Set $f_{ij}{}^{n+1} = f_{ij}{}^n + \lambda\left(g_{ij}{}^n - f_{ij}{}^n\right)$ for each road segment.

Step 6: If $\frac{\sqrt{\Sigma(f_{ij}{}^{n+1} - f_{ij}{}^n)^2}}{\Sigma f_{ij}{}^n} < \epsilon$ ($\epsilon = 0.005$ in the presented work), terminate the calculation, otherwise $n = n + 1$ and return to Step 2.

References

1. Frank M, Wolfe P (1956) An algorithm for quadratic programming. Naval Research Logistics Quarterly 3: 95.
2. LeBlanc LJ, Morlok EK, Pierskalla WP (1975) An efficient approach to solving the road network equilibrium traffic assignment problem. Transportation Research 9(5): 309-318.
3. Dijkstra EW (1959) A note on two problems in connexion with graphs. NumerischeMathematik 1: 269–271.
